# An Unhappy Subscription Method

**Published:** 1916-09-23

**Authors:** 


### AN UNHAPPY SUBSCRIPTION METHOD.

Appeals addressed to the sympathies and to the pockets of the public are just now so numerous that inevitably there has grown up a rivalry and competition between them. The effects of these influences have on the whole been stimulating, and much ingenuity and initiative have been aroused by them. Charity, equally with commerce, is all the better for the pressure which keeps the mind alert and prompts to new departures. Yet it is necessary to remember that enterprise has its limits, and that even for charity's sake sound and wise business methods must not be treated with despite. This common-sense counsel, at least so it seems to us, has been neglected in the appeal recently issued on behalf of the Cripples' Hospital and College at Alton. For the charity itself neither ourselves nor anyone else has anything but praise and good will. To convert crippled and helpless children into useful and self-supporting citizens is a work which touches the hearts of all; and the conspicuous success which has attended both the organisation and the medical treatment of the patients of this institution is widely recognised, by none more willingly than by ourselves. Even for the purposes of so excellent a charity, however, prudent and wise methods ought to be observed, and we think they are not observed when members of the public are asked to give promissory notes involving the donation of stated sums " on the declaration of peace." There is, it is true, a sentimental quality in the phrase which is not without its attraction, and when the happy date arrives many may well wish to celebrate it with a thankoffering which very reasonably may take shape as a gift to some charitable organisation. But the date is an entirely unknown quantity, and neither the public nor the private circumstances of the hour can be foreseen. For individuals to give undertakings to pay given sums of money under such conditions is not in accordance with sound economic principles, and a committee which urges such a step has not been happily inspired. The bond will fall due no one knows when or how, and thus the guarantee may require translation at some highly inconvenient moment.

The committee of the Alton Hospital justify this form of appeal on the ground that, without the promises desired, they will not be able to arrange plans for the reorganisation of the institution immediately on the termination of the war. To us, such a reason appears both inadequate and unsound. The committee cannot, so we are told, enter on the new scheme until the sum necessary for completion is assured. The promissory notes, we venture to say, will not afford them this assurance. In circumstances easy to be imagined many of the sums thus promised could not be collected, and therefore the notes do not afford a sound and secure business basis on which to rest present and future expenditure. When peace actually arrives the Alton Hospital may fairly claim its due meed of attention, but to ask a guarantee which is to be translated into performance on an uncertain date and in unknown circumstances is a financial method which is unwise for subscribers to accept and insecure as a justification for new enterprises.

